# Factors Associated with Smoking and Drinking among Early Adolescents in Vanuatu: A Cross-Sectional Study of Adolescents and Their Parents

**DOI:** 10.3390/ijerph17228412

**Published:** 2020-11-13

**Authors:** Emi Nakaseko, Sayaka Kotera, Minato Nakazawa

**Affiliations:** Department of Public Health, Graduate School of Health Sciences, Kobe University, Kobe 654-0142, Japan; skotera@port.kobe-u.ac.jp (S.K.); minato-nakazawa@people.kobe-u.ac.jp (M.N.)

**Keywords:** early adolescents, tobacco smoking, alcohol drinking, parents/guardians, peers, Vanuatu

## Abstract

This cross-sectional study determined whether various factors, such as parental behavior, attitude, and knowledge and sibling and peer behaviors, were associated with smoking and drinking among early adolescents in the Republic of Vanuatu. For this purpose, logistic regression analysis was used to determine the relative importance of the factors as well as the influences of the parents/guardians, siblings, and peers. The participants consisted of 157 seventh- and eighth-grade adolescents (mean age = 13.3 years; 52.2% girls), including their parents/guardians, from three public schools in Vanuatu. According to the results, the proportions of smokers and drinkers among the adolescents were 12.7% each, while the majority of the parents/guardians disapproved of underage smoking and drinking. In addition, peer influences (i.e., regularly smoking and/or drinking and offering tobacco and/or alcohol) was significantly associated with ever smoking and drinking, whereas parental and sibling influences did not have a significant impact on ever smoking and drinking. In sum, being given tobacco or alcohol from peers had the strongest association with ever smoking and drinking among the adolescents in this study. Thus, future school-based intervention programs should focus on enhancing early adolescents’ life skills, including the ability to resist offers of tobacco and/or alcohol from their peers.

## 1. Introduction

Harmful tobacco and alcohol use, two of the four most common modifiable behavioral risk factors for major noncommunicable diseases (NCDs), are serious health burdens, especially in low- and middle-income countries (LMICs) [1,2]. In particular, the Pacific island countries (PICs), most of which are LMICs [3], are home to the highest population of NCD deaths in the world [4]. NCDs are also an important driver of premature (age < 70 years) deaths in the majority of the PICs, with rates measurably higher than lower-middle-income global averages [5]. The Republic of Vanuatu, classified as a lower-middle-income country by the World Bank [3], also faces increasing rates of NCDs and premature mortality [6]. In fact, in 2016 alone, NCDs accounted for approximately 74% of all deaths in the country [1]. Meanwhile, tobacco use accounts for 17.8% of deaths among men and 7.3% among women; both of these percentages are higher than the average rates of death due to tobacco smoking for men and women in countries classified as medium by the Human Development Index [7]. Additionally, alcohol accounts for 20.2% and 14.5% of liver cirrhosis for men and women, respectively, in Vanuatu [2].Although some PICs have made progress in controlling tobacco use and improving physical activity levels during the past decade, strengthening taxation-based measures and conducting key evidence-based interventions to reduce tobacco use and alcohol consumption are still significant challenges in most PICs [8]. Thus, it is important to consider the health burdens of harmful tobacco and alcohol use to reduce the number of premature NCD deaths in this Pacific island nation.

In general, both early (12–14 years old) and late (15–17 years old) adolescence are critical risk periods for the initiation of substance and alcohol use [9]. Globally, approximately 12% of adolescents 13–15 years of age are current smokers [10], while 26.5% of adolescents 15–19 years of age are current drinkers [2]. Moreover, protecting early adolescents from exposure to smoking and drinking should be an important public health priority, since 90% of adults who are regular daily smokers stated that their first use of cigarettes occurred before the age of 18 [11]. As for alcohol consumption, adolescents are currently increasing their alcohol use and becoming heavy drinkers as young adults [12]. In fact, the 2016 Vanuatu Global School-based Student Health Survey (GSHS) of adolescents (13–15 years of age) showed that 27.5% of boys and 14.9% of girls had used some tobacco product, while 15.4% of boys and 9.7% of girls had drunk alcohol [13]. These percentages were significantly higher than those in the 2011 Vanuatu GSHS [14]. As a result, the Vanuatu NCD Policy and Strategic Plan 2016–2020 was introduced to raise awareness about the dangers of tobacco use and the harmful consumption of alcohol among the youth in the country [15]. However, evidence-based prevention programs for school-aged students have yet to progress. Unless measures are taken, the consumption rate of tobacco and alcohol among adolescents will continue to increase, which, in turn, may contribute to a country-wide rise in NCDs.

Although various factors at the personal (behavioral/mental health, neurological developments, etc.), micro (families, schools, and peers), and macro (socioeconomic and physical environments) levels contribute to harmful substance use among adolescents [9], parental and peer influences have been the subjects of focus in numerous studies [16,17,18,19,20,21,22,23,24,25,26,27,28,29,30,31,32,33]. Thus, it is necessary to take the developmental characteristics of early adolescents into account when considering the reasons why they are more likely to be influenced by their parents and peers. In this regard, one systematic review indicated that since the value systems of early adolescents change from being mostly defined by their parents to being more strongly influenced by their peers, they place greater emphasis on the latter [34]. Conversely, one study indicated that early adolescents generally receive social support from both their parents and friends [35]. Taken together, early adolescence tends to be the stage in which peer influences gradually increase, whereas parental influences decrease. Yet, both parents and peers still have a significant influence on early adolescents’ decision-making and behavior.

Some studies that investigated family and peer influences on smoking and drinking among early adolescents indicated that their smoking and drinking habits were influenced by those of their parents, siblings, and peers, although peer influences were the strongest [16,17,18,19,20]. Moreover, three longitudinal studies indicated that friends’ smoking and drinking behaviors had larger magnitude of association with those of adolescents compared with their parents’ or siblings’ behaviors, [21,22,23], while some studies found that the effect of friends’ smoking and drinking behaviors was stronger among early adolescents than among middle or late adolescents [23,24,25]. Conversely, two studies found that the magnitude of the parental effect was relatively stable from early through late adolescence [24,25]. These findings demonstrate that the effect of peer smoking and drinking behaviors on early adolescents tends to be stronger than the parental effect. On the contrary, one longitudinal study indicated that peer smoking only predicted the early onset of smoking, however, parental smoking predicted both the onset and level of smoking [26]. Another study indicated that the parental habit of consuming tobacco, alcohol, and close friend’s substance use behavior or peer pressure influenced the alcohol intake of adolescents [27].

With respect to the parental effect, some studies investigated the influence of parental monitoring, parental styles, and parent–adolescent communication on substance use [28,29,30,31,32,33]. Longitudinal studies indicated that lower parental monitoring was associated with increasing onset of tobacco and alcohol in adolescents [28,29]. Other studies indicated that parent–adolescent communication regarding substance use, including the negative effects of substance, was significantly associated with lower level of tobacco and alcohol use [30,31]. Furthermore, studies that examined the influence of parenting practice and styles related to substance use indicated that child exposure to family member(s) substance use and positive parental norms about alcohol were associated with increased child substance use [32]; in addition, the mother’s stringent behaviors were significantly associated with a decrease in the risk of smoking and drinking [33]. Findings from previous studies [16,17,18,19,20,21,22,23,24,25,26,27,28,29,30,31,32,33] highlighted the fact that parents and peers appear to be the key influencing factors in early adolescents’ smoking and drinking initiation behaviors. Therefore, it is necessary to discuss these factors to protect early adolescents from initiation of tobacco and alcohol use.

Although many studies have examined the parental and peer influences on smoking and drinking behaviors among early adolescents, limited studies have focused on LMICs [21,27,36] and PICs [37]. Our previous study [38], which investigated the relationship between students’ (sixth to eighth grade; 12–14 years of age) and family members’ smoking and drinking behaviors in Vanuatu, showed that the students’ school grades, gender and the family members’ smoking habits were significantly associated with the students’ ever smoking. Meanwhile, their attitudes toward drinking and perceptions of food consumption were significantly associated with the students’ ever drinking [38]. However, we could not identify the parental and peer influences on the students’ smoking and drinking behaviors. Moreover, few studies have investigated such influences on the smoking and drinking behaviors of early adolescents in Vanuatu, with limited research on the attitudes and knowledge regarding smoking and drinking among their parents/guardians.

Therefore, the present study determined whether various factors, such as parental behavior, attitude, and knowledge and sibling and peer behaviors, were associated with tobacco and alcohol use among early adolescents in Vanuatu. Overall, the data regarding the smoking and drinking behaviors of the adolescents and the perceived smoking and drinking behaviors of their siblings and peers were obtained from the adolescents’ reports. As for the parental factors, the data were obtained from the parents’ reports, but their perceived behaviors and attitudes were obtained from the adolescents’ reports. It is hoped that the findings will not only contribute to the development of evidence-based intervention programs for preventing underage smoking and drinking in Vanuatu, but they will be used as a reference for other LMICs and PICs.

## 2. Materials and Methods

### 2.1. Study Design and Procedures

This cross-sectional study was conducted in March 2019. The target population of the study was seventh- and eighth-grade adolescents and their parents/guardians in Vanuatu. In this case, a parent/guardian was defined as a man/woman who lives with and takes care of the adolescent, but does not need to be the biological parent. According to the Vanuatu Ministry of Education and Training, 9856 students studied in grades 7and 8 in the whole Vanuatu in 2019 [39]. This study was conducted in cooperation with the Japan International Cooperation Agency (Vanuatu Office), the Vanuatu Ministry of Health, the Vanuatu Ministry of Education and Training, and the Shefa Provincial Education Office. Convenience sampling was used to identify and recruit three public schools as the sample. The schools were also selected on the basis of the advice and information from the Shefa Provincial Education Office that was responsible for the public schools in the Efate islands and the Vanuatu Ministry of Education and Training. Among the three sample schools, two were from the capital city of Port Vila, and one was from a rural area in the Efate islands. One school in Port Vila and another school in the rural area are the primary schools, called center schools, that educate students from grades 1–8. Although Vanuatu has six years of compulsory primary education, the center schools provide eight years of education. Another sample school from Port Vila is a combined primary and secondary school. Written informed consent was obtained from the principals of the three schools prior to the survey.

The data from both the adolescents and their parents/guardians were obtained by using self-administered questionnaires. The same reference number was assigned to each pair of questionnaires to align the data. However, the researchers were blinded to these numbers and other personal information. This study was approved by the Ethics Committee of Himeji Dokkyo University (Approval Number: 18–11) and the Vanuatu Ministry of Health Executive Committee (Approval Number: DPH02/2-LT/mt). The parents/guardians were also provided with a written explanation regarding their participation along with their minor-aged adolescents in this survey. The parents/guardians who participated in the survey were given a ballpoint pen and toothbrush as compensation for participating. The study procedures were carried out in accordance with principles in Declaration of Helsinki.

The study participants were asked to complete the questionnaires in their respective classrooms. A researcher visited each classroom, distributed the questionnaires to the participants, and provided information about the purpose of the study, voluntary participation, confidentiality, and anonymity.

Regarding the parents/guardians, they were asked to complete the questionnaires in their homes. More specifically, one questionnaire for the parents/guardians was distributed to each seventh- and eighth-grade adolescent. The parents with more than two children in either grade only completed one questionnaire about their oldest child. Moreover, they were informed about the purpose of the study, voluntary participation, confidentiality, and anonymity through a written explanation. The completed questionnaires were returned to the researcher by the adolescents themselves. The submission of the questionnaires was treated as consent to participate in the survey.

### 2.2. Sample

Data analysis comprised 157 seventh- and eighth-grade adolescents (mean age = 13.3 years; 52.2% girls) and corresponding parents/guardians from three public schools in Vanuatu. In total, 336 pairs of questionnaires were distributed, after which 336 questionnaires were obtained from the adolescents and 221 were obtained from the parents/guardians (response rate 65.8%). Thus, 221 initial data sets were used in this study. However, 157 data sets were included, (valid response rate 71.0%) based on the following inclusion criteria: the adolescent reported that he/she had ever or never used tobacco/alcohol; and the parent/guardian reported that both or either of them had ever used tobacco/ alcohol or both of them have never used tobacco/ alcohol. Among the 64 excluded respondents (30 and 34 male and female adolescents, respectively, and their parents/guardians), 28 reported missing data on adolescents, whereas 36 was because of missing data on parents/guardians.

### 2.3. Measures

The dependent variables in this study were ever smoking and ever drinking among the adolescents, while parental, sibling, and peer factors were the independent variables. The study considered parental behavior, attitude, knowledge and sibling and peer behaviors regarding smoking and drinking as influencing factors of ever smoking and drinking among adolescents. The study adapted and modified the GSHS [40] and Global Youth Tobacco Survey [41] questionnaires in line with its objectives. The questions on the smoking and drinking behaviors of the adolescents and their parents/guardians as well as the level of parental involvement were based on the Vanuatu GSHS [40]. As for the questions on the smoking-related knowledge of the parents/guardians, they were based on the Global Youth Tobacco Survey [41]. Other measures, such as parental approval of underage smoking and drinking, parental attitude related to adolescent’s health, parental knowledge and attitude related to alcohol and marijuana, were originally introduced by the study.

A pilot study was undertaken in March 2018 to test the methodology and develop the questions for the formal study. A total of 31 adolescents (aged 12–14 years) and 12 of their parents/guardians from one public school in the Efate islands in Vanuatu were included in the pilot study. The adolescents and their parents/guardians were required to complete the questionnaire in a classroom. The average time to complete the questionnaire was 30 and 20 min for the adolescents and parents/guardians, respectively. During the survey, few adolescents found difficulty in understanding that sibling means a brother or sister, whereas the parents/guardians did not require clarification regarding the questionnaire. Based on the pilot study, minor revisions and improvements were made to the original questionnaire (e.g., “sibling” was changed to “brother or sister”). The questionnaire for the formal survey was then considered complete and usable.

#### 2.3.1. Adolescents

The adolescents were first asked to provide their age and gender. For the subsequent analyses, they were asked whether they had ever experimented with smoking/drinking and whether they have smoked tobacco/drank alcohol in the past 30 days (currently smoke tobacco/drink alcohol). To assess the level of parental involvement, the adolescents were asked whether their parents/guardians understood their problems and worries, gave them advice and guidance, and had open communication with them. To assess the perceived parental attitudes, the adolescents were asked whether their parents/guardians had ever offered them tobacco/alcohol. Finally, to assess the effects of sibling and peer factors, the adolescents were asked about the current smoking/drinking habits of their siblings and peers, and whether they had ever offered the adolescents tobacco/alcohol.

#### 2.3.2. Parents/Guardians

The parents/guardians were first asked to provide their age, gender, and work status as well as their respective smoking/drinking habits (i.e., such habits of the fathers/male guardians and those of the mothers/female guardians). For the subsequent analyses, the ever and current smoking/drinking categories were divided into “Both or either” or “Neither” for the parents/guardians who ever/currently used tobacco/alcohol. As for their work status, it was divided into “Both or either” or “Neither” for the parents/guardians who had a regular occupation.

To assess the parental approval of underage smoking/drinking, the parents/guardians were asked whether they allowed their underage adolescents to smoke/drink. To assess the parental attitudes toward smoking/drinking, the parents/guardians were asked the following: if they ever talked with their adolescents about the health hazards of tobacco, alcohol, and marijuana; and if they ever made their adolescents purchase tobacco and alcohol. To assess the parental attitudes toward their adolescent’s health, the parents/guardians were asked if they were aware of their adolescent’s health, both mentally and physically. Finally, to assess the parental knowledge and attitude related to substance use, the following items were included: the health hazards of tobacco smoking; the health hazards of second-hand smoke; the difficulty of stopping smoking; and the health hazards of alcohol and marijuana use.

### 2.4. Data Analysis

The statistical analysis was performed by using SPSS Version 20 for Windows (IBM, Armonk, NY), with a significance level of *p* < 0.05, while a chi-square test was performed to examine the bivariate associations between each dependent variable (parental, sibling and peer factors) and ever smoking and drinking among the adolescents. Fisher’s exact test was also used as the sample size was small with ≤5 in a cell, while logistic regression analysis (adjusted for the gender and residential area of the adolescents) was performed to determine the factors associated with ever smoking and drinking among the adolescents. The variables that showed significant differences in each bivariate analysis were considered as independent variables in a separate logistic regression analysis for ever smoking and drinking. Moreover, the variable entry criterion in the statistical model was set at *p* < 0.05 (using the backward stepwise selection method), while the results were demonstrated as odds ratio (OR) and 95% confidence intervals (CIs).

## 3. Results

### 3.1. Characteristics of the Study Sample

Among the 157 adolescents in this study, 75 (47.8%) were boys and 82 (52.2%) were girls. The mean age of the adolescents was 13.3 years, while the proportions of ever smokers and drinkers were 12.7% each, and those of current smokers and drinkers were 3.9% and 3.3%, respectively. More than half (55.3%) and almost half (49.3%) of the adolescents had friends who smoked tobacco and drank alcohol, respectively. It should be noted that 5.8% of the adolescents were offered tobacco or alcohol by their parents/guardians (see Table 1).

Among the parents/guardians, the proportion of both or either of them was as follows: ever smokers (47.1%); current smokers (33.8%); ever drinkers (69.4%); and current drinkers (30.8%). In addition, the majority of the parents/guardians disapproved of underage smoking and drinking. However, 12.1% previously made their adolescent purchase tobacco, despite the fact that the sale of tobacco products to individuals under 18 years of age is prohibited in Vanuatu [42]. More than 80% of the parents/guardians had talked about the health hazards of tobacco and alcohol with their adolescents, while more than 90% were aware of the health hazards of tobacco, second-hand smoke, marijuana, and harmful alcohol use. Furthermore, 34% were unaware of the difficulty of stopping smoking (see Table 2).

### 3.2. Bivariate Analysis

Table 3 presents the bivariate associations between the parental, sibling, and peer factors and smoking and drinking experience among the adolescents. Among the adolescents, those whose parents/guardians (both or either of them) currently consumed alcohol, those who were ever offered tobacco or alcohol by their parents/guardians, siblings or peers, and those who had peers who smoked tobacco or drank alcohol showed a higher prevalence of ever smoking, compared to the adolescents with contrasting situations. Moreover, the adolescents who lived in an urban area, who had peers who smoked tobacco or drank alcohol, who had ever been offered tobacco or alcohol by their peers, and who had siblings who smoked tobacco showed a higher prevalence of ever drinking, compared to the adolescents with contrasting situations.

Bivariate analysis indicated that parental, sibling and peer factors were significantly associated with ever smoking among the adolescents, whereas sibling and peer factors instead of parental factors were significantly associated with ever drinking among the adolescents.

### 3.3. Multivariate Analysis

Table 4 includes the results of the forward stepwise logistic regression analysis, with ever smoking and ever drinking as the dependent variables, and gender and residential area as the control variables. In the logistic regression analysis for ever smoking, the following variables were considered as independent variables: parental drinking; peer smoking and drinking; and being offered tobacco or alcohol by parents/guardians, siblings or peers. The results showed that tobacco or alcohol offered by peers (OR = 37.54; 95% CI, 7.81–180.46; *p* < 0.001) and peer drinking (OR = 8.41; 95% CI, 1.55–45.54; *p* < 0.05) were significantly associated with increased ever smoking.

In the logistic regression analysis for ever drinking, the following variables were considered as independent variables: sibling smoking; peer smoking and drinking; and being offered tobacco or alcohol by peers. The results showed that being offered tobacco or alcohol by peers (OR = 6.19; 95% CI, 1.49–25.78; *p* < 0.05) and peer smoking (OR = 5.91; 95% CI, 1.26–27.71; *p* < 0.05) were significantly associated with increased ever drinking.

## 4. Discussion

Many studies have indicated that parental and peer factors are the key influencing factors in early adolescents’ smoking and drinking initiation and behavior. However, limited studies have focused on LMICs and PICs such as Vanuatu. Thus, this study determined whether various factors, such as parental behavior, attitude, and knowledge and sibling and peer behaviors, were associated with smoking and drinking among early adolescents in Vanuatu. The key finding of this study was that ever smoking and drinking among early adolescents should not only be interpreted by investigating parental influences, but also peer influences. In the case of parental influences, parental alcohol drinking and parental offer of tobacco or alcohol had bivariate associations between ever smoking among the adolescents. However, these factors did not show a significant association in the forward stepwise logistic regression analysis. Overall, the findings suggest that it is necessary to focus on peer influences to create more effective intervention programs for preventing early adolescents from engaging in underage smoking and drinking in Vanuatu.

### 4.1. Peer Influences

The logistic regression analysis showed that having friends who smoke or drink, and having peers who offer tobacco or alcohol were significantly associated with ever smoking and drinking among the adolescents. Previous studies have indicated that peer influences on adolescents’ smoking and drinking behaviors were stronger, compared to those of their parents and siblings [16,17,18,19,20], while longitudinal studies indicated that friends’ smoking and drinking behaviors had a larger magnitude of association with those of adolescents compared with their parents’ or siblings’ behaviors [21,22,23]. The findings of the present study are in line with these studies, meaning that significant peer influences on the adolescents’ smoking and drinking behaviors were found.

Previous studies have also examined peer influences on adolescents’ risk behaviors [43,44,45]. According to one study on risk-taking and risky decision-making among adolescents and adults, adolescents were more prone to risk when in peer groups, compared to adults [43]. Another study that investigated the relationship between peer pressure and the risk behaviors of students aged 13–15 showed that having friends who were involved in the same risk behaviors was the dominant factor influencing risky behaviors [44]. In addition, one study indicated that more than 60% of students obtained their first cigarette from their peers (63%) [45], while one systematic review on the predictors of smoking onset in adolescents showed a positive correlation between friends’ smoking and smoking onset in 26 out of 28 studies [46]. Altogether, these findings [43,44,45,46] indicated that peer influences were among the most dominant factors in smoking and drinking among early adolescents. It is important to note that the majority of the studies that indicated the significant effect of peer influences were conducted in upper-middle- and high-income countries (UMICs) [16,17,18,19,20,22,23,24,25,26,43,44,45]. However, even though Vanuatu is classified as an LMIC, the results provide vital evidence regarding the effect of peer influences on smoking and drinking among early adolescents in the country.

### 4.2. Parental Influences

Some studies have indicated that although parental influences were less influential, compared to peer influences, they were significantly linked with adolescents’ smoking and drinking behaviors [16,17,18,19,20,21,22,23]. Related studies have also indicated that parental monitoring, parental styles, and parent–adolescent communication predicted the substance use of adolescents [28,29,30,31,32,33]. However, unlike these studies [16,17,18,19,20,21,22,23,28,29,30,31,32,33], we could not find a significant link between the factors of behavior, attitude, and knowledge about smoking and drinking among the parents/guardians and ever smoking and drinking among the adolescents. In other words, the results of the present study did not support the hypothesis.

Previous studies have also found no significant link between smoking and drinking behaviors among parents and their adolescents. For instance, parental smoking was positively related to the smoking onset of adolescents in 16 out of 23 studies [46]. This finding suggests that more than 30% of the studies did not find a significant link between parental smoking and the smoking onset of their adolescents. Meanwhile, another study [47] that targeted early adolescents (mean age = 12.3 years) and their parents found that less-restrictive parental rules and attitudes about alcohol were associated with the use of alcohol by the adolescents. However, parental alcohol use did not play a significant role, since the gap between parental roles and early adolescence might be too large to model the effects of drinking [47]. This also suggests that parents might not be a viable role model for early adolescents around 12 years of age [47]. Although the present study’s sample was limited to 12–14-year-olds, the findings suggest that their peers were viable role models for smoking tobacco and drinking alcohol, instead of their parents/guardians.

Another possible explanation for the lack of association between parental influences and ever smoking and drinking in adolescents may be attributed to the traditional culture and customs in Vanuatu. For example, in Vanuatu, it is traditional for the household unit and children to rely on family, community, and kinship networks, as in other PICs [48]. Moreover, there is a high level of social cohesion in Vanuatu, with much respect and trust given to the village chiefs and Christian church leaders [49]. Another study stated that for the children, there are many caregivers, and parenting can be performed by other relatives and adults in Pacific culture including Vanuatu; in ni-Vanuatu, “it takes a whole village to raise a child” is a popular belief [50]. Such customs in Vanuatu lead us to presume that other individuals (e.g., village chiefs and church leaders) and even the neighborhoods/communities themselves are actively engaged in the upbringing of early adolescents in Vanuatu. In this regard, previous studies have highlighted the association between neighborhood/community influences and tobacco and alcohol use among adolescents [51,52,53]. More specifically, higher neighborhood/community disorganization and less social cohesion were associated with a higher level of adolescent alcohol, tobacco, and drug use [51]. In related studies, neighborhood/community cohesion had a limited direct effect on adolescent delinquency and alcohol use [52], while protective neighborhood/community factors were correlated with low alcohol, tobacco, and drug use [53]. Thus, the finding of the present study that no significant effect from parental influences was observed might be attributed to the fact that many neighborhoods/communities, including the parents/guardians, were engaged in the education and protection of early adolescents and their peers. However, further research is necessary to clarify any direct/indirect influences of neighborhoods/communities in discouraging smoking and drinking among early adolescents.

An additional explanation for the lack of parental influences may be attributed to methodological limitations, such as sample selection bias and a lack of statistical significance, due to the relatively small sample size. In this regard, this study’s sample showed that the rates of never smokers were significantly higher among the adolescents whose parents/guardians participated in the study (n = 221), compared to the adolescents whose parents/guardians were not involved in this study (n = 125). Since the sample of parents/guardians was comprised of those who voluntarily answered the questionnaires (response rate 65.8%), this study included a sample selection bias. It is also possible that those with negative attitudes toward the reduction of underage smoking and drinking were excluded, while the parents/guardians with positive attitudes and their adolescents were included in this study. Interestingly, only a number of parents/guardians reported that they approved of underage smoking or drinking, they were unaware of any health hazards from tobacco and alcohol, and they did not discuss any health hazards with their children. Thus, it is possible to conclude that there was no statistically significant link between parental influences and ever smoking and drinking among the adolescents in this study.

### 4.3. Implications

The results indicated that tobacco or alcohol offered by peers was the most dominant predictor of ever smoking and drinking among the adolescents. According to the WHO, schools are prime locations for NCD prevention, especially through life skills education, and they can provide supportive healthy environments for training children in the acquisition of life skills [54]. Moreover, the WHO suggested that it is necessary for adolescents to enhance their negotiation/refusal and conflict management skills to help them reject invitations from friends to engage in smoking and drinking, and by doing so, it could possibly lead to an overall reduction in tobacco and alcohol use throughout their school lives [54]. In sum, the present study provides sufficient evidence to conclude that school-based life skills education is essential for reducing underage smoking and drinking in Vanuatu.

It should be noted that although teachers play an important role in school-based life skills education, the roles of parents/guardians and neighborhoods/communities in discouraging underage smoking and drinking must not be ignored. In this regard, previous studies have investigated the relationship between parental influence and friendship choices among adolescents. For instance, one longitudinal study indicated that parental involvement and a healthy family relationship helped adolescents make better choices when forming friendships and peer groups, while the parents and families had an indirect influence on substance use later in their lives [55]. Another study indicated that active parental involvement helped stop the use of alcohol, tobacco, and drugs among adolescents as well as prevented them from making poor friend choices [56]. Although this study did not investigate parental involvement and monitoring in adolescents’ peer selection, parental roles in protecting adolescents from making poor friend choices and accessing smoking or drinking was found in previous studies. Finally, as mentioned earlier, some studies have demonstrated the link between neighborhood/community factors and tobacco and alcohol use among adolescents [51,52,53]. Since high neighborhood/community cohesion is found in Vanuatu [48,49,50], neighborhoods/communities can play an essential role in preventing early adolescents from smoking and drinking. Therefore, it is necessary to discuss the roles of parents/guardians, neighborhoods/communities, and teachers when establishing intervention policies aimed at preventing underage smoking and drinking in Vanuatu.

### 4.4. Limitations and Strengths

The study has its limitations. First, given that the findings are based on a limited sample size due to convenience sampling, the results should be interpreted with caution. The relatively small sample size may have weakened the statistical significance of the influential factors of smoking tobacco and drinking alcohol among the early adolescents. Moreover, the low OR and 95% CI were most likely attributed to the small sample size. Second, selection bias was noted in the sample. In fact, the study was only conducted in three public schools on one island, despite Vanuatu being an archipelago that comprised approximately 80 islands. Out of these islands, 65 are inhabited. Furthermore, as previously mentioned, the sample of parents/guardians only included those who voluntarily answered the questionnaires. Thus, parents/guardians with positive attitudes and their adolescents might be included in this study. Because of these limitations (limited sample size and selection bias), our findings might not be representative the whole of Vanuatu. Therefore, future studies should include a sample size that covers a greater number of inhabited islands to generalize the findings. Third, the study collected data only through self-reported questionnaires. As such, the questions may have been misunderstood or acquiescence bias may have occurred, especially among the adolescents who might have been hesitant to answer questions about their smoking and drinking behaviors, given that both activities are considered illegal among adolescents aged less than 18 years in Vanuatu [42,57].

Despite these limitations, the present study has several strengths. First, to the best of our knowledge, no previous study has clarified the link between peer influence and smoking and drinking behaviors among early adolescents in Vanuatu. The findings suggest that a stronger focus on peer influence is necessary to implement more robust intervention programs for preventing smoking and drinking among early adolescents. Second, the results provide vital evidence that peer influence is one of the dominant influencing factors of smoking and drinking among early adolescents in LMICs and UMICs. Third, the study highlights the importance of education on school-based life skills among adolescents in PICs and LMICs, including Vanuatu. The study hopes that the results can be used as a reference for the formulation of effective intervention programs for preventing early adolescents from underage smoking and drinking, not only in Vanuatu but also in other PICs and LMICs.

## 5. Conclusions

This cross-sectional study determined whether various factors, such as parental behavior, attitude, knowledge, and sibling and peer behaviors, were associated with smoking and drinking among early adolescents in Vanuatu. Based on the results, peer influences (instead of parental influences) played a significant role in ever smoking and drinking among the early adolescents in this study. The implication of the findings is that future school-based intervention programs aimed at reducing smoking and drinking should focus on enhancing adolescents’ life skills, including the ability to reject tobacco or alcohol from their peers. Despite the limitations, this study provides additional evidence linking the influential role of peers in the initiation of smoking and drinking among early adolescents in both LMICs and UMICs. Further research is required to determine the factors associated with smoking and drinking among early adolescents in PICs, LMICs, and Vanuatu and should facilitate school-based policies aimed at reducing tobacco and alcohol use among early adolescents.

## Figures and Tables

**Table 1:** Characteristics of the sample of adolescents (n = 157).

Variable	n	%
**Residential area**		
Urban	108	68.8
Rural	49	31.2
**Gender**		
Boy	75	47.8
Girl	82	52.2
**Age**		
Mean ± SD	13.3 ± 1.01	
**Ever smoked tobacco**		
Yes	20	12.7
No	137	87.3
**Currently smokes tobacco**		
Yes	6	3.9
No	147	96.1
**Ever drunk alcohol**		
Yes	20	12.7
No	137	87.3
**Currently drinks alcohol**		
Yes	5	3.3
No	147	96.7
**Parental offer of tobacco or alcohol**		
Yes	9	5.8
No	147	94.2
**Parental involvement** (those who answered “Yes”)		
Parents/guardians usually understand my problems and worries.	125	80.1
Parents/guardians usually provide advice and guidance.	147	94.2
Parents/guardians usually have open communication with me.	143	91.1
**Sibling smoking**		
Yes	40	25.5
No	117	74.5
**Sibling drinking**		
Yes	48	30.8
No	108	69.2
**Sibling offer of tobacco or alcohol**		
Yes	10	6.4
No	146	93.6
**Peer smoking**		
Yes	83	55.3
No	67	44.7
**Peer drinking**		
Yes	73	49.3
No	75	50.7
**Peer offer of tobacco or alcohol**		
Yes	17	10.9
No	139	89.1

Note: The total excludes the non-responses. Currently smokes tobacco: smoked any tobacco product in the past 30 days. Currently drinks alcohol: drank alcohol in the past 30 days.

**Table 2:** Characteristics of the sample of parents/guardians (n = 157).

**Variable**	**n**	**%**
**Responses to the questionnaires**		
Fathers and mothers (male and female guardians) together	69	43.9
Fathers/male guardians alone	45	28.7
Mothers/female guardians alone	39	24.8
No answer	4	2.5
**Age** (Mean ± SD)		
Father/male guardians	43.5 ± 8.18	
Mothers/female guardians	39.0 ± 7.02	
**Having a regular occupation**		
Both or either of the parents/guardians	129	89.0
Neither of the parents/guardians	16	11.0
**Ever smoked tobacco**		
Both or either of the parents/guardians	74	47.1
Neither of the parents/guardians	83	52.9
**Currently smokes tobacco**		
Both or either of the parents/guardians	52	33.8
Neither of the parents/guardians	102	66.2
**Ever drunk alcohol**		
Both or either of the parents/guardians	109	69.4
Neither of the parents/guardians	48	30.6
**Currently drinks alcohol**		
Both or either of the parents/guardians	44	30.8
Neither of the parents/guardians	99	69.2
**Approval of underage smoking**		
Approval	20	13.7
Disapproval	126	86.3
**Approval of underage drinking**		
Approval	21	15.0
Disapproval	119	85.0
**Parental attitude related to smoking and drinking** (those who answered “Yes”)		
Ever talked about the health hazards of tobacco with the adolescent.	125	83.3
Ever talked about the health hazards of alcohol with the adolescent.	132	88.0
Ever talked about the health hazards of marijuana with the adolescent.	138	92.0
Ever made the adolescent purchase tobacco.	18	12.1
Ever made the adolescent purchase alcohol.	4	2.7
**Parental attitude related to the adolescent’s health** (those who answered “Yes”)		
Conscious about the adolescent’s health, both mentally and physically.	145	97.3
**Parental knowledge and attitude related to substance use** (those who answered “I think so”)		
Smoking tobacco causes cancer, lung disease, heart disease, and stroke.	151	98.7
Someone has started smoking, so it will be difficult to quit.	101	66.0
The smoke from other people’s tobacco smoke is harmful to you as well.	151	98.7
Drinking too much alcohol can cause mental and behavioral problems.	139	90.8
Breathing marijuana can damage the brain and nerves.	147	96.7

Note: The total excludes the non-responses. Currently smokes tobacco: smoked any tobacco product in the past 30 days. Currently drinks alcohol: drank alcohol in the past 30 days.

**Table 3:** Bivariate association between parental, sibling, and peer factors, and ever smoking and drinking among adolescents (n = 157).

Variable	Smoking Status of the Adolescents					Drinking Status of the Adolescents				
	Ever-Smoker		Never-Smoker		*p*-Value	Ever-Drinker		Never-Drinker		*p*-Value
	n	%	n	%		n	%	n	%	
**Demographic Factors**										
**Residential area**										
Urban	17	15.7	91	84.3	0.094	19	17.6	89	82.4	0.004
Rural	3	6.1	46	93.9		1	2.0	48	98.0	
**Adolescents’ gender**										
Girl	10	12.2	72	87.8	0.831	12	14.6	70	85.4	0.456
Boy	10	13.3	65	86.7		8	10.7	67	89.3	
**Parental Factors**										
**Having a regular occupation**										
Both/either of the parents/guardians	17	13.2	112	86.8	0.377	17	13.2	112	86.8	0.121
Neither of the parents/guardians	1	6.3	15	93.8		0	0.0	16	100.0	
**Ever smoked tobacco**										
Both/either of the parents/guardians	11	14.9	63	85.1	0.451	7	9.5	67	90.5	0.245
Neither of the parents/guardians	9	10.8	74	89.2		13	15.7	70	84.3	
**Currently smokes tobacco**										
Both/either of the parents/guardians	8	15.4	44	84.6	0.527	6	11.5	46	88.5	0.703
Neither of the parents/guardians	12	11.8	90	88.2		14	13.7	88	86.3	
**Ever drunk alcohol**										
Both/either of the parents/guardians	15	13.8	94	86.2	0.384	12	11.0	97	89.0	0.327
Neither of the parents/guardians	5	10.4	43	89.6		8	16.7	40	83.3	
**Currently drinks alcohol**										
Both/either of the parents/guardians	10	22.7	34	77.3	0.045	7	15.9	37	84.1	0.658
Neither of the parents/guardians	10	10.1	89	89.9		13	13.1	86	86.9	
**Approval of underage smoking**										
Approval	3	15.0	17	85.0	0.502	2	10.0	18	90.0	0.538
Disapproval	16	12.7	110	87.3		16	12.7	110	87.3	
**Approval of underage drinking**										
Approval	3	14.3	18	85.7	0.530	2	9.5	19	90.5	0.513
Disapproval	15	12.6	104	87.4		15	12.6	104	87.4	
**Talking about the health hazards of tobacco with the adolescent**										
Yes	17	13.6	108	86.4	0.349	17	13.6	108	86.4	0.349
No	2	8.0	23	92.0		2	8.0	23	92.0	
**Talking about the health hazards of alcohol with the adolescent**										
Yes	16	12.1	116	87.9	0.406	17	12.9	115	87.1	0.594
No	3	16.7	15	83.3		2	11.1	16	88.9	
**Talking about the health hazards of marijuana with the adolescent**										
Yes	19	13.8	119	86.2	0.364	18	13.0	120	87.0	0.534
No	0	0.0	12	100.0		1	8.3	11	91.7	
**Ever made the adolescent purchase tobacco**										
Yes	5	27.8	13	72.2	0.057	3	16.7	15	83.3	0.704
No	14	10.7	117	89.3		16	12.2	115	87.8	
**Ever made the adolescent purchase alcohol**										
Yes	1	25.0	3	75.0	0.424	0	0.0	4	100.0	0.576
No	18	12.4	127	87.6		19	13.1	126	86.9	
**Conscious about the adolescent’s health**										
Yes	18	12.4	127	87.6	0.424	18	12.4	127	87.6	0.424
No	1	25.0	3	75.0		1	25.0	3	75.0	
**Having knowledge about the health hazards of smoking**										
Yes	20	13.2	131	86.8	0.755	19	12.6	132	87.4	0.245
No	0	0.0	2	100.0		1	50.0	1	50.0	
**Having knowledge about the difficulty of stopping smoking**										
Yes	12	11.9	89	88.1	0.543	14	13.9	87	86.1	0.686
No	8	15.4	44	84.6		6	11.5	46	88.5	
**Having knowledge about the second-hand smoke**										
Yes	19	12.6	132	87.4	0.245	19	12.6	132	87.4	0.245
No	1	50.0	1	50.0		1	50.0	1	50.0	
**Having knowledge about the health hazards of alcohol use**										
Yes	16	11.5	123	88.5	0.09	18	12.9	121	87.1	0.574
No	4	28.6	10	71.4		2	14.3	12	85.7	
**Having knowledge about the health hazards of marijuana**										
Yes	18	12.2	129	87.8	0.129	19	12.9	128	87.1	0.511
No	2	40.0	3	60.0		1	20.0	4	80.0	
**Parental offer of tobacco or alcohol**										
Yes	4	44.4	5	55.6	0.017	2	22.2	7	77.8	0.324
No	16	10.9	131	89.1		18	12.2	129	87.8	
**Parental understanding of problems and worries**										
Yes	13	10.4	112	89.6	0.069	17	13.6	108	86.4	0.405
No	7	22.6	24	77.4		3	9.7	28	90.3	
**Parental advice and guidance**										
Yes	19	12.9	128	87.1	0.68	19	12.9	128	87.1	0.301
No	1	11.1	8	88.9		0	0.0	9	100.0	
**Having open communication with the parents/guardians**										
Yes	18	12.6	125	87.4	0.56	19	13.3	124	86.7	0.442
No	2	14.3	12	85.7		1	7.1	13	92.9	
**Sibling Factors**										
**Sibling smoking**										
Yes	7	17.5	33	82.5	0.295	9	22.5	31	77.5	0.032
No	13	11.1	104	88.9		11	9.4	106	90.6	
**Sibling drinking**										
Yes	8	16.7	40	83.3	0.338	8	16.7	40	83.3	0.338
No	12	11.1	96	88.9		12	11.1	96	88.9	
**Sibling offer of tobacco or alcohol**										
Yes	5	50.0	5	50.0	0.004	3	30.0	7	70.0	0.12
No	15	10.3	131	89.7		17	11.6	129	88.4	
**Peer Factors**										
**Peer smoking**										
Yes	17	20.5	66	79.5	0.001	18	21.7	65	78.3	0.001
No	2	3.0	65	97		2	3.0	65	97.0	
**Peer drinking**										
Yes	17	23.3	56	76.7	<0.001	15	20.5	58	79.5	0.014
No	2	2.7	73	97.3		5	6.7	70	93.3	
**Peer offer of tobacco or alcohol**										
Yes	12	70.6	5	29.4	<0.001	6	35.3	11	64.7	0.01
No	8	5.8	131	94.2		14	10.1	125	89.9	

Note: The total number excludes non-responses. The chi-square test was used to examine the bivariate association between each dependent variable and ever smoking and drinking among adolescents. Fisher’s exact test was used in the case of a sample size (with 5 ≤ in a cell).

**Table 4:** Factors associated with ever smoking and ever drinking among the adolescents through logistic regression analysis (forward stepwise).

Variable	Ever Smoking				Ever Drinking			
	OR	95% CI	*p*-Value		OR	95% CI	*p*-Value	
Peer offer of tobacco or alcohol								
Yes	37.54	7.81–180.46	<0.001	***	6.19	1.49–25.78	0.012	*
No	1				1			
**Peer drinking**								
Yes	8.41	1.55–45.54	0.013	*				
No	1							
**Peer smoking**								
Yes					5.91	1.26–27.71	0.024	*
No					1			

*** *p* < 0.001, * *p* < 0.05. OR = odds ratio; 95% CI = 95% confidence interval. Note: Logistic regression and the forward stepwise selection method were used with the adolescents’ gender and residential area as control variables. The variables in which significant differences were observed in bivariate analysis were considered as the independent variables.
